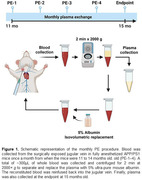# Plasma Exchange reduces amyloid plaques and microglial activation in APP/PS1 mice at the advanced stage of pathology

**DOI:** 10.1002/alz.095636

**Published:** 2025-01-09

**Authors:** Suelyn Koerich, Santiago Ramirez, Natalia Astudillo, Claudio Soto

**Affiliations:** ^1^ The University of Texas Health Science Center at Houston, Houston, TX USA

## Abstract

**Background:**

Amyloid plaques predominantly consist of extracellular aggregates of amyloid‐β (Aβ) peptide and represent a hallmark pathological feature of Alzheimer’s disease (AD). Evidence suggests that peripheral Aβ plays a crucial role in AD pathogenesis. Additionally, studies have indicated that Plasma Exchange (PE) with albumin replacement can slow the decline or stabilize AD symptoms. However, the factors that influence the mechanisms behind this are largely unknown. In this context, we have previously demonstrated the potential benefit of PE in reducing Aβ plaques in APP/PS1 mice at the early stage of AD pathology. Here, we tested the effectiveness of using PE monthly to decrease Aβ accumulation and alter brain inflammation in APP/PS1 mice at the advanced stage of pathology.

**Methods:**

300µL of blood was obtained monthly from APP/PS1 mice from the jugular vein. Subsequently, the blood was centrifuged to remove the plasma, and the same volume was replaced with 5% albumin in saline and reinfused into the jugular vein (Figure 1). The procedure was performed four times between 11 and 14 months, and animals were humanely euthanized at 15 months. For histological analysis, brains were dissected after transcardiac brain perfusion. The right hemisphere was fixed for histology, and 10µm sections were stained with thioflavin‐S (Thio‐S) to analyze burden, number of plaques, and plaques size or incubated with anti‐Aβ antibody 4G8 plus Iba‐1 to analyze Aβ burden and microglia area. All images were analyzed using NIH Image J software.

**Results:**

The data demonstrate that PE with a 5% mouse albumin solution effectively reduced thioflavin‐S‐positive amyloid burden and plaque size in the cortex and Aβ burden in the cortex and hippocampus. Additionally, the Iba‐1^+^ area in the hippocampus was significantly lower in PE‐treated animals than in controls.

**Conclusions:**

These results suggest that PE reduced amyloid plaques applied at advanced stages of the disease and supports PE’s potential efficacy as an alternative non‐pharmacological intervention for attenuating the progression of AD pathology.